# Is globalization healthy: a statistical indicator analysis of the impacts of globalization on health

**DOI:** 10.1186/1744-8603-6-16

**Published:** 2010-09-17

**Authors:** Pim Martens, Su-Mia Akin, Huynen Maud, Raza Mohsin

**Affiliations:** 1 International Centre for Integrated assessment and Sustainable development (ICIS), Maastricht University, P.O. Box 616, Maastricht, The Netherlands; 2 Department of Sustainability Sciences, Leuphana University, Lüneburg, Germany

## Abstract

It is clear that globalization is something more than a purely economic phenomenon manifesting itself on a global scale. Among the visible manifestations of globalization are the greater international movement of goods and services, financial capital, information and people. In addition, there are technological developments, more transboundary cultural exchanges, facilitated by the freer trade of more differentiated products as well as by tourism and immigration, changes in the political landscape and ecological consequences. In this paper, we link the Maastricht Globalization Index with health indicators to analyse if more globalized countries are doing better in terms of infant mortality rate, under-five mortality rate, and adult mortality rate. The results indicate a positive association between a high level of globalization and low mortality rates. In view of the arguments that globalization provides winners and losers, and might be seen as a disequalizing process, we should perhaps be careful in interpreting the observed positive association as simple evidence that globalization is mostly good for our health. It is our hope that a further analysis of health impacts of globalization may help in adjusting and optimising the process of globalization on every level in the direction of a sustainable and healthy development for all.

## Introduction

In the past, globalization has often been seen as a more or less economic process characterized by increased deregulated trade, electronic communication, and capital mobility. However, globalization is becoming increasingly perceived as a more comprehensive phenomenon that is shaped by a multitude of factors and events, and that is reshaping our society rapidly; it encompasses not only economic, political, and technological forces, but also social-cultural and environmental aspects. This increased global economic integration, global forms of governance, and globally inter-linked social and environmental developments are often referred to as globalization. However, depending on the researcher or commentator, globalization is interpreted as growing integration of markets and nation-states and the spread of technological advancements [1]; receding geographical constraints on social and cultural arrangements [2]; the increased dissemination of ideas and technologies [3]; the threat to national sovereignty by trans-national actors [4]; or the transformation of the economic, political and cultural foundations of societies [5]. In our view, globalization is an overarching process encompassing many different processes that take place simultaneously in a variety of domains (e.g., governance structures, markets, communication, mobility, cultural interactions, and environmental change). The pluralistic definition of globalization by Rennen and Martens [6] offers a conceptualization capturing the complexity of different dimensions;, processes; scale-levels; and linkages and pathways; characterizing the relationship between globalization and health. Hence, contemporary globalization is defined as the intensification of cross-national interactions that promote the establishment of trans-national structures and the global integration of cultural, economic, ecological, political, technological and social processes on global, supra-national, national, regional and local levels [6].

Looking at the health of populations, Martens [7] and Huynen [8], amongst others, argue that changes in drivers of disease are brought about not only by economic changes, but also by changes in the social, political, and environmental domains at local, regional, and global levels. Health improvements experienced in developed countries over the past centuries are mainly vested in social and environmental changes, whereas more recent health improvements in developing countries can be broadly related to knowledge transfer and socio-cultural determinants. Nowadays, global processes influence all these important health determinants. Hence, globalization and its underlying processes have brought about vast changes in both health determinants and related health outcomes. As a result, the geographical scale of important health issues is significantly increasing [9]. The link between global mobility and the spread of infectious diseases is perhaps the best-known health effect of globalization. However, it is only one of the many possible health implications of globalization. Many scholars have tried to conceptualize the possible linkages between globalization and health. Woodward et al. [10], for example, propose a framework based on three component circular processes of globalization: openness; cross-border flows; and rules and institutions. However, their conceptualization mainly focused on the health effects of economic globalization. Labonte and Torgerson [11] review different conceptualizations of the globalization-health relationship, resulting in a diagrammatical synthesis that mainly focuses on governmental policy changes as well as economic determinants of health, but with the inclusion of an environmental pathway. Hence, many of these approaches primarily emphasize the economic and institutional side of globalization, defining globalization in a rather narrow way. Labonte and Schrecker [12,13] took a somewhat different approach in their framework for the Commission of Social Determinants of Health, conceptualizing how globalization affects disparities in access to social determinants of health.

Because of the multitude of underlying processes shaping the globalization-health link, ideas about globalization, health determinants and possible outcomes should be broadened. The causality of human health is multi-factorial and many population health problems are invariably embedded in a global context [8]. Taking this broader view on globalization and global health, Huynen et al. [9] developed an integrated conceptual framework for the health implications of globalization. We can conclude that a variety of both negative and positive effects are expected to influence our health in the (near) future [8,9] (see Table 1 for examples), but it is still very uncertain what the overall health outcomes will be. Academic literature shows an ongoing polarized debate [14]. The limited empirical evidence on the multiple links between globalization and health poses a problem [15]. Many scholars urge for elaboration and possible quantitative evidence to support the hypothesized relationships [9,10,14-21]. In this paper we try to answer the question if the process of globalization improves the health of populations (or not).

**Table 1:** Positive and negative health impacts of globalization: some examples ([8,9].

Positive health impacts	Negative health impacts
-Diffusion of knowledge and technologies, improving health services;	-Spread of infectious diseases due to increased movement of goods and people;
-Diffusion of knowledge and technologies, improving food and water availability (e.g. irrigation technology);	-Spread of unhealthy lifestyles due to, for example, cultural globalization, global trade and marketing;
-Improvements in health care or sanitation due to economic development;	-Brain drain in the health sector;
-Global governance efforts, such as WHO's Framework Convention on Tobacco Control (WHO FCTC) and WHO's Global Outbreak Alert and Response Network;	-Health risks due to global environmental change;
-Increased access to affordable food supplies due to free trade.	-Decreased government spending on public services due to, for example, Structural Adjustment Programmes (SAPs);
	-Inequitable access to food supplies due to asymmetries in the global market.

## Methodology

In this paper we use an indicator-based approach [22] linking the Maastricht Globalization Index (MGI) (a measure of globalization) to important health indicators, correcting for possible confounding factors. The MGI as well as the selected health indicators and confounders will be discussed in the following sections. Subsequently, the performed statistical analyses will be clarified.

### The Maastricht Globalization Index

**Figure 1:**
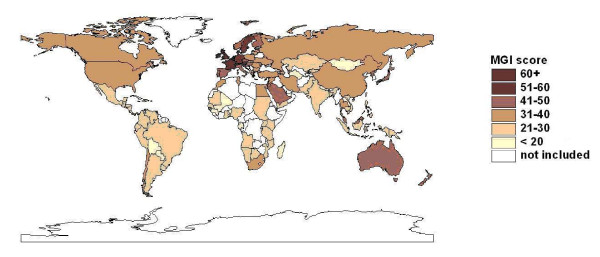
**Map of the Maastricht Globalization Index (MGI) 2008 **[27].

In this section, we briefly describe the Maastricht Globalization Index (MGI) [22]. The MGI was developed by Martens and Zywiets [23] and Martens and Raza [24] to improve upon existing globalization-indices. The need for a balance between broad coverage, data availability and quality motivated the following choice of indicators (see Table 2), with data for 117 countries (see Figure 1).

**Table 2:** Maastricht Globalization Index (MGI) variables [23,24].

Category	Variable name	Variable definition
Political Domain	Embassies	Absolute number of in-country embassies and high commissions
	Organizations	Absolute number of memberships in international organizations
	Military	Trade in conventional arms as a share of military spending
Economic domain	Trade	Imports + exports of goods and services as a share of GDP
	FDI	Gross foreign direct stocks as a share of GDP
	Capital	Gross private capital flows as a share of GDP
Social & Cultural Domain	Migrants	Those who changes their country of usual residence per 100 inhabitant
	Tourism	International arrivals + departures per 100 inhabitants
Technological Domain	Phone	Incoming + outgoing international telephone traffic in minutes per capita
	Internet	Internet users as a share of population
Ecological Domain	Eco footprint	Ecological deficit in global ha

The MGI is constructed in a four-stage process (see also [25]). The first stage is conceptual and choices are made about which variables are most relevant and should be included in the index. In the second stage, suitable quantitative measures are identified for these variables. In the third stage, following [26], each variable is transformed to an index with a zero to one hundred scale (this differs from earlier calculations constructing the MGI [23]). Higher values denote more globalization. The data are then transformed--on the domain level--according to the percentiles of the base year (2000) distribution (using the formula ((V_i _- V_min_)/(V_max _- V_min_) × 100). In the last and final stage, a weighted sum of the measures is calculated to produce the final score, which is then used to rank and compare countries. The "most globalized" country has the highest score. Within each domain, every variable is equally weighted. The MGI scores are simply added, *i.e.*, all domains receive the same weight. In this paper, we use the MGI calculated for 2008 [27].

Several limitations in using the MGI (and in general globalizations indices) exist. Since there are missing data on the share of international linkages that are regional rather than global, it is impossible to distinguish globalization from internationalisation and regionalisation with complete certainty. Therefore, there is an underlying assumption that countries with many international links have a correspondingly greater number of global linkages. As expected, international statistics on eleven different indicators ranging from politics and military to the environment have widely varying degrees of data quality, reflecting the different capabilities and priorities of the organisations collecting the data. Of particular concern are the domains in which the underlying data have not been collected by official international bodies like the World Bank, IMF and/or other UN organizations, but by private or semi-public organisations. In addition, many countries are reluctant to share information about activities related to their national security, which creates data gaps that are not easily filled.

The fact that countries with fewer international linkages tend to publish less data and are less likely to be included in international statistics biases against states that are less globalized [28]. Additionally, despite being members of the UN and most other international bodies, countries with totalitarian or communist regimes (e.g., North Korea, Cuba) are often excluded in international financial statistics. Therefore, this also leads to their exclusion due to lack of data. Finally, yet importantly, countries that are too small to collect internationally coherent statistics and/or are strongly integrated into the economies of their big neighbours (e.g., Luxembourg, Monaco, and Swaziland) are also missing from the statistics and therefore excluded from the MGI.

Both the sensitivity to extreme values and year-to-year variations are a major concern for the robustness of other indices for globalization. With the methodology used to construct the MGI, the sensitivity of the index to extreme values has been sharply reduced since the distribution is now centred on the mean of a component rather than just lying somewhere between the extreme values. Similarly, the strongest year-to-year variations are filtered by the averaging process for the highly volatile components, sharply decreasing the dependence on the choice of base year in some of the component indicators. Furthermore, several weighting methods for composite indicators--like the MGI--exist, all with their own pros and cons. Regardless which weighting method is used, weights are in essence value judgments. For maximum transparency, we have relied on equal weighting [29]. Next, we have tested the sensitivity of the weighting scheme at the domain level. With respect to the weights for the five domains tested in the sensitivity analysis, the country rankings are consistent for approximately half of the countries. The allocation of the weights must be evaluated with care according to its analytical rationale, globalization relevance, and implied value judgments.

### Health Indicators

In order to link the extent that a country is globalized with the status of population health in a country, several indicators for mortality have been selected, based on the World Health Statistics [30]:

• Infant mortality rate (per 1000 live births, both sexes): "[...] the probability of a child born in a specific year or period dying before reaching the age of one, if subject to age-specific mortality rates of that period [31]".

• Under-five mortality rate (probability of dying by age 5 per 1000 live births, both sexes): "the probability of a child born in a specific year or period dying before reaching the age of five, if subject to age-specific mortality rates of that period [31]".

• Adult mortality rate (probability of dying between 15 to 60 years per 1000 population, both sexes): "probability that a 15-year-old person will die before reaching his/her 60th birthday [31]".

According to the World Health Organization [31], indicators representing such mortality rates provide an accurate view of overall population health. The infant mortality rate and under-five mortality rate are principal indicators used to assess child health, and overall health and development in a country [32]. The WHO uses these indicators to measure progress on the Millennium Development Goals [31-33]. Low levels of life expectancy are inherently related to higher levels of child mortality. The adult mortality rate has become a widely used indicator for assessing the overall patterns of mortality in a country's population. The growing importance of this indicator is particularly stressed by the increasing disease burden from non-communicable diseases among adults (economically productive age categories) by ageing trends and health transitions [32]. The selected mortality indicators are available for all 117 countries in the MGI-indicator dataset.

### Confounding factors

The relationship between the process of globalization (MGI) and the selected health outcomes cannot be isolated from other, possibly related developments. Therefore, possible confounding factors in the MGI-health relationship have been identified based on existing literature: income level and income growth (often represented by GDP per capita; GNP per capita; or Growth of GDP per capita) [7,34,35]; water quality [35]; Health expenditures and financing [34,35]; Smoking [34] secondary education [35]; and availability of public health resources (such as vaccinations) [35]. Table 3 provides an overview of the selected indicators associated with these confounding factors (including sample size, year and source).

**Table 3:** Overview of selected confounders

Indicator	Definition	n (sample size)	Year(s)	Source
GDP per capita growth (annual%)*	"Annual percentage growth rate of GDP per capita based on constant local currency. GDP per capita is gross domestic product divided by midyear population. GDP at purchaser's prices is the sum of gross value added by all resident producers in the economy plus any product taxes and minus any subsidies not included in the value of the products. It is calculated without making deductions for depreciation of fabricated assets or for depletion and degradation of natural resources (The World Bank Group, 2010)"	114	2008	World DataBank, World Development Indicators and Global Development Finance [36]
Prevalence of undernourishment (% of population)	"[...] the percentage of the population whose food intake is insufficient to meet dietary energy requirements continuously. Data showing as 2.5 signifies a prevalence of undernourishment below 2.5% (The World Bank Group, 2010)."	116	2006	World Databank, World Development Indicators and Global Development Finance [36]
Total expenditure on health as a percentage of gross domestic product	"Level of total expenditure on health (THE) expressed as a percentage of gross domestic product (GDP) (WHO, 2009a)."	117	2006	WHO [30,31]
Health expenditure, public (% of GDP)	"Public health expenditure consists of recurrent and capital spending from government (central and local) budgets, external borrowings and grants (including donations from international agencies and nongovernmental organizations), and social (or compulsory) health insurance funds (The World Bank Group, 2010)."	117	2007	World Databank, World Development Indicators and Global Development Finance [36]
Health expenditure, total (% of GDP)	"Total health expenditure is the sum of public and private health expenditure. It covers the provision of health services (preventive and curative), family planning activities, nutrition activities, and emergency aid designated for health but does not include provision of water and sanitation (World Bank Group, 2010)."	117	2007	World Databank, World Development Indicators and Global Development Finance [36]
Literacy rate, adult total (% of people ages 15 and above)	"Adult literacy rate is the percentage of people ages 15 and above who can, with understanding, read and write a short, simple statement on their everyday life (World Bank Group, 2010)."	97	2000-2008**	World Databank, World Development Indicators and Global Development Finance [36]
Total enrolment, primary (% net) 2000-2008	"Total enrollment is the number of pupils of the school-age group for primary education, enrolled either in primary or secondary education, expressed as a percentage of the total population in that age group (World Bank Group, 2010)."	109	2000-2008**	World Databank, World Development Indicators and Global Development Finance [36]
School enrolment, secondary (% net)	"Net enrollment ratio is the ratio of children of official school age based on the International Standard Classification of Education 1997 who are enrolled in school to the population of the corresponding official school age. Secondary education completes the provision of basic education that began at the primary level, and aims at laying the foundations for lifelong learning and human development, by offering more subject- or skill-oriented instruction using more specialized teachers (World Bank Group, 2010)."	94	2000-2008**	World Databank, World Development Indicators and Global Development Finance [36]
Total fertility rate (per woman)	"The average number of children a hypothetical cohort of women would have at the end of their reproductive period if they were subject during their whole lives to the fertility rates of a given period and if they were not subject to mortality. It is expressed as children per woman (WHO, 2009a)."	117	2006	WHO [30,31]
Smoking prevalence, females (% of adults)	"[...] the percentage of women ages 15 and over who smoke any form of tobacco, including cigarettes, cigars, and pipes, and excluding smokeless tobacco. Data include daily and non-daily smoking (World Bank Group, 2010)."	95	2006	World Databank, World Development Indicators and Global Development Finance [36]
Improved water source (% of population with access)	"[...] the percentage of the population with reasonable access to an adequate amount of water from an improved source, such as a household connection, public standpipe, borehole, protected well or spring, and rainwater collection. Unimproved sources include vendors, tanker trucks, and unprotected wells and springs. Reasonable access is defined as the availability of at least 20 liters a person a day from a source within one kilometer of the dwelling (World Bank Group, 2010)."	107	2000-2006**	World Development Indicators and Global Development Finance (World Bank Group 2010)
Improved sanitation facilities (% of population with access)	"Access to improved sanitation facilities refers to the percentage of the population with at least adequate access to excreta disposal facilities that can effectively prevent human, animal, and insect contact with excreta. Improved facilities range from simple but protected pit latrines to flush toilets with a sewerage connection. To be effective, facilities must be correctly constructed and properly maintained (World Bank Group, 2010)."	102	2000-2006**	World Development Indicators and Global Development Finance [36]
Immunization, DPT (% of children ages 12-23 months)	"Child immunization measures the percentage of children ages 12-23 months who received vaccinations before 12 months or at any time before the survey. A child is considered adequately immunized against diphtheria, pertussis (or whooping cough), and tetanus (DPT) after receiving three doses of vaccine (World Bank Group, 2010)."	116	2008	World Development Indicators and Global Development Finance [36]
Immunization, measles (% of children ages 12-23 months)	"Child immunization measures the percentage of children ages 12-23 months who received vaccinations before 12 months or at any time before the survey. A child is considered adequately immunized against measles after receiving one dose of vaccine (World Bank Group, 2010)."	116	2008	World Development Indicators and Global Development Finance [36]

* Other GDP measures (including GDP per capita (PPP)) have not been included for the following reasons: a) the GDP measure shows multicollinearity with the other confounders and/or b) the GDP measure when tested does not function as a confounder in the MGI-health indicator relationship.

** Data for most recent year available in this range has been selected for each country. It should be noted that all compiled datasets largely exist of data stemming from the latest years that the set covers, and only few cases from earlier years have been added to meet the sampled countries in the MGI dataset. Confounders that did not have any or much current data available for the sampled countries did not qualify for a compilation of data over several years, and were therefore not included in this study.

Many other possible confounders have been considered for this analysis, but could not be included for different reasons. A large group of confounders have been excluded based on lack of data availability for the sampled countries, and/or a lack of current data.^i ^Other variables could not be selected for this study because when tested not all criteria for confounding could be met. ^ii^

### Statistical methods and analysis

Correlation analysis has been conducted as a first step, in order to obtain the crude associations between the indicators used. For this we applied the non-parametric Spearman's correlation analyses, as not all variables showed a normal distribution [37]^iii^.

Next, least squares (LS) simple linear regression analysis has been performed to gain an insight in the possible associations between the MGI and the mortality indicators, as well as the strength of these associations for each of the underlying MGI Domains (all without controlling for possible confounding). Subsequently, LS multiple linear regression analysis has been performed, in order to assesses if and to what extent the MGI can explain a proportion of the variance in the dependent variables 'infant mortality rate'; 'under-five mortality rate'; and 'adult mortality rate'; whilst controlling for the selected confounding factors [38]. It has been tested whether the models meet the regression model assumptions and are not subject to outliers [38-40]^iv^. Based on the results, a transformation of the mortality indicators into a natural logarithm (Ln) was required for a proper regression analyses.

To construct the final multiple regression models, backward step-wise linear regression has been used. For this process, the correlation coefficients between the dependent/confounding variables and the independent variables have been used as a criterion to prioritize the different confounding variables for inclusion in the model (i.e. variables showing a higher correlation coefficient with the independent variable have precedence over variables showing lower correlation coefficients). Moreover, the correlation coefficients have been used to identify possible cases of multicollinearity between the dependent and confounding variables. Here, the common threshold of not having a correlation coefficient higher than 0.80 has been applied [38]. When a possible case of multicollinearity has been detected, one of the two variables involved has not been included in the model, where the variable with the lower Spearman's correlation with the dependent variable has been excluded over the other variable. During the step-wise backward linear regression, the R-square and the F-statistic (as a test for the global usefulness of the model) have been used to determine the final model [38,39]^v^. All analyses have been performed in SPSS 15.0.

## Results

### Results Spearman correlation

To give an indication of the crude associations between the MGI, and the MGI Domains, with the health indicators, the Spearman's correlations are given in Table 4.

**Table 4:** Spearman's correlations between the Maastricht Globalization Index (MGI); the MGI Domains; and the mortality indicators.

*n = 117*	Infant mortality rate 2007	Under-five mortality rate 2007	Adult mortality rate 2007
**MGI 2008**	-.798*	-.803*	-.717*

**MGI domains**			

Political 2008	-.440*	-.445*	-.487*
Economic 2008	-.421*	-.428*	-.270*
Social & cultural 2008	-.706*	-.712*	-.556*
Technological 2008	-.891*	-.892*	-.805*
Ecological 2008	-.397*	-.400*	-.390*

*Significant at the 0.01 level (2-tailed).

The results show that the MGI has a statistically significant^vi ^negative correlation (at α = 0.01) with all selected mortality indicators (-0.798, -0.803, -0.717, respectively). When taking a closer look at the individual domains of the MGI, the results in Table 4 reveal that all underlying domains have a significant negative correlation (at α = 0.01) with the mortality indicators. The correlations between the mortality rates and the socio-cultural, and technological domains are particularly strong.

### Results simple linear regression models

**Figure 2:**
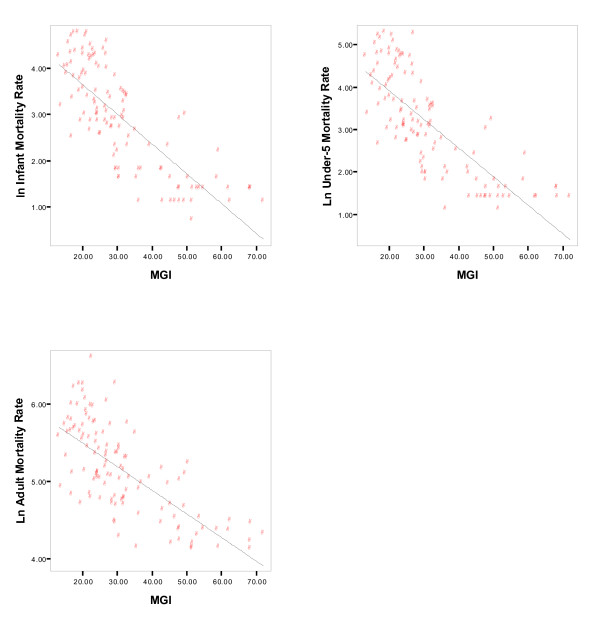
**Scatterplots and linear regression between the Maastricht Globalization (MGI) and the selected mortality indicators**.

Tables 5 and 6 and Figure 2 show the simple linear regression outcomes of the mortality indicators (Ln transformed) with the MGI and the MGI Domains, respectively, as dependent variables; without correction for confounding factors The associations between the MGI/MGI Domains and the mortality indicators suggested by the Spearman's correlation outcomes logically correspond with the associations that can be ascertained from these univariate regression analyses. All results are significant (at α = 0.01) in the expected direction. From the R-squares, it follows that the variation in the MGI partly explains the variation in all mortality indicators. Similar to the correlation results, the R-squares in Table 6 indicate that the 'social & cultural' and the 'technical' domains of the MGI show a stronger association with the mortality indicators.

**Table 5:** Linear regression coefficients (β) for the Maastricht Globalization Index (MGI) and selected mortality indicators.

*n = 117*	*Ln *Infant mortality rate 2007	*Ln *Under-five mortality rate 2007	*Ln *Adult mortality rate 2007
Constant (β_0_)	4.941*	5.263*	6.103*
MGI 2008 (β_1_)	-.064*	-.067*	-.030*
R-square	.616	.596	.502

* Significant at the 0.01 level (2 tailed)

**Table 6:** Linear regression coefficients (β) for the Maastricht Globalization Index (MGI) domains and selected mortality indicators.

*n = 117*	*Ln *Infant mortality rate 2007	*Ln *Under-five mortality rate 2007	*Ln *Adult mortality rate 2007
Constant (β_0_)	3.752*	4.021*	5.609*
Political 2008 (β_1_)	-.024*	-.026*	-.013*
R-square	.217	.210	.237

Constant (β_0_)	3.506*	3.772*	5.362*
Economic 2008 (β_1_)	-.030*	-.031*	-.011*
R-square	.178	.177	.090

Constant (β_0_)	3.491*	3.748*	5.406*
Social & Cultural 2008 (β_1_)	-.037*	-.038*	-.016*
R-square	.400	.388	.294

Constant (β_0_)	3.744*	4.003*	5.542*
Technological 2008 (β_1_)	-.039*	-.041*	-.019*
R-square	.667	.633	.551

Constant (β_0_)	3.978*	4.272*	5.676*
Ecological 2008 (β_1_)	-.017*	-.018*	-.008*
R-square	.085	.085	.077

* Significant at the 0.01 level (2 tailed)

### Results multiple regression models

Table 7, 8, and 9 show the results of the multiple regression models for Ln Infant mortality rate, Ln Under-five morality rate, and Ln Adult mortality rate. Overall, it can be observed that the R-squares are higher in all instances, in comparison to the results of the simple linear regression analyses in Table 5. This indicates that the models for all three mortality indicators have been improved in explanatory power by adding the confounding factors.

**Table 7:** Final regression model of the Ln Infant mortality rate; controlling for confounding factors.

Number of countries (n)	R-Square	F-statistic	Significance F-test
*76*	.880	130.544	.000
	Regression coefficient β	t-statistic	Significance t-test
Constant (β_0_)	7.142	13.875	.000
MGI 2008 (β_1_)	-.022	-4.539	.000
School enrollment, secondary (%net) 2000-2008 (β_2_)	-.021	-6.454	.000
Health Expenditure, public (% of GDP) 2007 (β_3_)	-.131	-3.725	.000
Total enrollment, primary (% net) 2000-2008 (β_4_)	-.018	-2.700	.009

**Table 8:** Final regression model of the Ln Under-five mortality rate; controlling for confounding factors.

Number of countries (n)	R-Square	F-statistic	Significance F-test
80	.885	144.099	.000
	Regression coefficient β	t-statistic	Significance t-test
Constant (β_0_)	7.469	14.126	.000
MGI 2008 (β_1_)	-.026	-5.922	.000
School enrollment, secondary (% net), 2000-2008 (β_2_)	-.024	-7.021	.000
Smoking prevalence, females (% of adults) 2006 (β_3_)	-.019	-3.506	.001
Total enrollment, primary (% net) 2000-2008 (β_4_)	-.019	-2.781	.007

For all three models, the confounders 'Total expenditure on health as a percentage of gross domestic product, 2006' and 'Health expenditure, total (% of GDP), 2007' were not included because of multicollinearity and conceptual overlap with 'Health expenditure, public (% of GDP) 2007'. Similarly, the confounder 'Immunization, DTP (% of children 12-23 months) 2008' has not been included in any of the models due to multicollinearity with 'Immunization, measles (% of children 12-23 months) 2008'.

### Multiple regression model for Infant mortality rate

For the model of Ln Infant mortality rate, the confounders 'Literacy rate, adult total (% of people ages 15 and above) 2000-2008'; 'Total fertility rate (per woman) 2006'; 'Improved water source (% of population with access) 2000-2006'; and 'Improved sanitation facilities (% of population with access) 2000-2006' were not included because of multicollinearity with 'School enrollment, secondary (% net) 2000-2008'. During the process of stepwise backward regression, the following confounders have been removed from the model based on an insignificant association with Ln Infant mortality rate (meaning a significance higher than α = 0.01) to create the final model: 'GDP per capita growth (annual%) 2008'; 'Immunization, measles (% children ages 12-23 months) 2008'; 'Prevalence of undernourishment (% of population) 2006'; and 'Smoking prevalence, females (% of adults) 2006'.

The results from final model of Ln Infant mortality rate (Table 7) shows significant t-values for all variables included. The coefficients for the MGI and the confounders all show the expected signs/direction. In addition, a high R-square (0.880) and a significant and high F-statistic is reached. The decrease in regression coefficients for the MGI compared to the results of the simple linear regression analysis indicates that the confounders play a significant role in the posed relationship. When controlling for the confounding factors, however, the MGI still remains significantly associated with the Ln Infant mortality rate.

### Multiple regression model for Under-five mortality rate

For the final model of Ln Under-five mortality rate (Table 8), the same confounders were excluded based on multicollinearity with 'School enrollment, secondary (% net) 2000-2008' as described for the previous model of Ln Infant mortality rate. During the process of stepwise backward regression, contrary to the model of Ln Infant mortality rate, 'Health expenditure, public (% of GDP) 2007' has been removed based an insignificant association with Ln Under-five mortality rate (higher than α = 0.01), but 'Smoking prevalence, females (% of adults), 2006' could be included in the final model.

The results from the final model (Table 8) show that all resulting coefficients display the expected signs, and all t-values are significant at the α = 0.01 level. The R-square is high (0.885) and the F-statistic is high and significant. The significance of the confounding factors indicates that these factors do play a relevant role in the relationship between the MGI and the Ln Under-five mortality rate. Hence, the higher MGI coefficient found for the simple linear regression might have been an overestimation of the association between the MGI and the Ln Under-five mortality rate, and this association has now been corrected for relevant confounding factors. When controlling for the confounding factors, however, the MGI still remains significantly associated with the Ln Infant mortality rate.

### Multiple regression model for Adult mortality rate

For the final model of Ln Adult mortality rate, the confounder 'School enrollment, secondary (% net) 2000-2008' has not been included in the model due to multicollinearity with 'Improved sanitation facilities (% of population with access) 2000-2006' (amongst other confounders). During the process of stepwise backward regression, all confounders^vii ^had to be eliminated from the model due to an insignificant association with the Ln Adult mortality rate (α = 0.01) except for 'Improved sanitation facilities (% of population with access) 2000-2006'. The insignificant associations of all other confounders with the Ln Adult mortality rate is a departure from what could be seen for the other models. This could be an indication that the selected confounders are not as relevant in the relationship between the MGI and the Ln Adult mortality rate.

The results from the final model (Table 9) show that all coefficients have the expected signs, and the t-values are significant (at α = 0.01). The R-square is relative high (0.612) and the F-statistic is significant. The decrease in regression coefficients for the MGI compared to the results of the simple linear regression analysis indicates that 'Improved sanitation facilities (% of population with access) 2000-2006' plays a significant role in the posed relationship. When controlling for this confounding factor, however, the MGI still remains significantly associated with the Ln Infant mortality rate.

**Table 9:** Final regression model of the Ln Adult mortality rate; controlling for confounding factors.

Number of countries (n)	R-Square	F-statistic	Significance F-test
*90*	.612	78.124	.000
	Regression coefficient β	t-statistic	Significance t-test
Constant (β_0_)	6.389	62.523	.000
MGI 2008 (β_1_)	-.012	-3.044	.003
Improved sanitation facilities (% of population with access) 2000-2006 (β_2_)	-.012	-7.069	.000

## Discussion

As this research focuses on indicators of mortality to highlight an important side of global health outcomes, it is interesting to look at some of the drivers directly related to mortality (or factors linking globalization and mortality) identified in the current body of research in this field. Martens [7] claims that increased income levels can result in a decrease in mortality rates, which ultimately impacts life expectancy rates positively. Burns, Kentor, and Jorgenson [35] focus on infant mortality and discuss a country's level of internal development and the related dependencies on the world economy (affecting domestic institutional structures) as a main driver. However, the level of a country's development and the resulting impact on infant mortality is not fully uncovered. Other factors they found to be related to infant mortality are the macro level effect of export commodity concentration, GDP per capita, health expenditures per capita, secondary education, and organic water pollution. They identified several mediating factors between global dependence and infant mortality: quality of water and health care, level of internal development such as GNP per capita, the role of ecology (pollution and misuse of land) as well as public health factors (lack of resources for public health can be seen with indicators such as scarcity of inoculation to childhood diseases, and the lack of trained medical personnel for pre-and post-natal care and for assistance with birth process itself) [35]

Cornia et al. [34] associate globalization mainly with economic changes, such as economy policy, protectionism, costs of technological transfer, privatization, market liberalization, trade and financial liberalization. Looking at the slow progress in infant mortality rates over the past decades, the authors suggest that many factors can be responsible for these slow improvements such as slow growth of household incomes, greater income volatility, shifts in health financing, amongst others. In this study, the effects of globalization are captured by comparing the timeframe of 1980-2000 (the era of globalization) with other timeframes, indicating changes in the following indicators: growth of GDP per capita, economic stability, income inequality, inflation and prices of basic goods, taxation and public health expenditure and health financing, migration and family arrangements, technical progress in health, smoking drinking and obesity, and random shocks [34].

The results of our analysis (Spearman's correlations, and simple and multiple linear regression analyses) indicate that the infant morality rate, under-five mortality rate and adult mortality rate all show a negative association with the process of globalization (as measured by the MGI). Specifically, technological globalization and socio-cultural globalization are shown to have strong associations with the selected health indicators. The multivariate analyses show that different confounders have been found to be significant in the three final models. Specifically, for Ln Infant morality rate confounders accounting for primary and secondary education and public health expenditures have been found to be significant. For the Ln Under-five mortality rate, next to the confounders for primary and secondary education, smoking prevalence under females have shown to be significant in the final model. Lastly, for the model of Ln Adult mortality rate, only a confounder on access to improved sanitation facilities has been significant. These factors, thus can possibly function as confounders in the relationships between the respective mortality rates with the MGI. However, the confounders in the final models could also be important mediating/causal factors in the association between the mortality rates and the MGI. Either way, in all multivariate models, the association between globalization and the mortality indicators remains significant after controlling for confounding factors.

Given the limited existing quantitative information on the association between globalization and health, the results might provide a crude initial indication of the potential advantageous effect of globalization on health. In view of the arguments that globalization provides winners and losers, and might be seen as a disequalizing process, we should perhaps be careful in interpreting the observed positive association between the MGI and health, as simple evidence that globalization is mostly good for our health. Important to note is that all indicators and data are on the country level, without a specific spatial dimension. Globalization interacts with health at levels that make measurement difficult, e.g., trans-border environmental issues, cultural transformations and a so-called 'global consciousness'. For example, the data do not show us that the most globalized countries might have lower mortality rates because they have exported their unhealthy pollution and other externalities of the production of goods and services they enjoy (and which contribute to their health) to people and environments in other parts of the world. Hence, some of the winners might be benefiting from their high levels of globalization at the expense of others. Importantly, it should also be noted that he MGI represents actual levels of globalization across different domains, rather then the mere implementation of neoliberal policies.

## Conclusion

In this paper, we consider the impact of the recent process of globalization on the health of populations. Looking at the results, globalization can be characterised as both more complicated and more surprising than was anticipated. One clear lesson can be learned from the many global assessments that have been produced over the past decades: dogmatic predictions regarding the earth's future are unreliable, ill-founded and misleading, and can be politically counterproductive. So, this analysis is beset with the uncertainties and assumptions that apply to any global statistical indicator analysis [41]. For example, if consumerism and global economic processes do have polluting and other unhealthy negative side-effects for some, it needs to be asked which direction these dynamics need to take for sustainable health for all. Furthermore, this analysis is based on 'present day data'. As the globalizing processes intensify over time, the indirect impacts of human-induced disruption of global biogeochemical cycles and global climate change, and their impacts on human health, may start to become more apparent [42,43]. Borghesi and Vecelli [44] also state that the available empirical evidence suggests that the current process of globalization is unsustainable in the long run unless we introduce new institutions and policies able to govern it, a similar claim being made by Tisdall [45] and Watanabe [46] looking at economic globalization only. Schrecker et al. [47] reject furthermore the presumption that globalization will yield health benefits as a result of its contribution to rapid economic growth and associated reduction in poverty.

Hence, for future research we hypothesize that a country performance might be classified into four categories (adopted from [48]: vicious cycle (low globalisation, high mortality), globalisation-lopsided (high globalisation, high mortality), health-lopsided (low globalisation, low mortality) or virtuous cycle (high globalisation, low mortality).

We hypothesize that a country performance might be classified into four categories (adopted from [48]: vicious cycle (low globalisation, high mortality), globalisation-lopsided (high globalisation, high mortality), health-lopsided (low globalisation, low mortality) or virtuous cycle (high globalisation, low mortality). In the vicious cycle, any efforts to properly integrate into the global process are yet unsuccessful, but might even result in (temporary) adverse health effects (e.g. Ghana). Globalization-lopsided may happen when integration into the globalization process has not yet resulted in major health benefits, or might have even resulted in increasing health problems (e.g. Egypt). Health-lopsided might happen, when health improvements occur that are not related to any globalization benefits, but due to other domestic polices or developments (e.g. Peru). In a virtuous cycle, countries might have benefited from their integration into the globalization process, while averting any associated health risks. It is important to note, however, that for some countries the virtuous cycle could be the result of bias due to causal sequence (i.e. did all the major improvement in health already occurred prior to the modern-day globalization process?) (e.g. the Netherlands).

Example countries:

• Vicious cycle (low globalization, high mortality): Since the 1980s, Ghana has implemented the macro-economic policies prescriptions and Structural Adjustment Programs of the Bretton Woods Institutions (BWI), but with limited success. The commitment to privatisation and cuts in public spending have, however, resulted in users fees in health care and, subsequently, to restricted access for the poor, especially in rural areas [49]. In the Upper Volta region, health care use is believed to have decreased by 50 percent [50]. An additional health problem is, for example, the out-migration of doctors and nurses [51]. Ghana has experienced an increase in adult mortality rate from 272 per 1000 population in 1990 to 331 per 1000 population in 2006 [30].

• Health-lopsided (low globalization, low mortality): Peru has experienced important health improvements in the past decades (although the gap between rich and poor remains a problem) [52] and in 1990, Peru's adult mortality rate had already declined to 178 per 1000 population [30]. Hence, many of Peru's health improvements occurred before President Fujimori started to push for integration into the global market via extensive macro-economic policies in the early 1990s. There has been macroeconomic growth since, but limited increase in development. In 2006, adult mortality rate had declined further to 136 per 1000 population [30], but Peruvians have a lower health status compared to the continental average and some are concerned about the possible adverse globalization impacts, such as increasing inequality and decreasing labor standards [53,54].

• Globalization-lopsided (high globalization, high mortality): Since the mid-1970s, Egypt has been going through a process of increasing integration into the world economy. Even though Egypt implemented further macro-economic policies and structural adjustment programs in the 1980s and 1990s, the associated impacts on economic growth and development have been disappointing and uneven [55], for example resulting in increasing unemployment. Egypt also faced many health challenges such as low formal health coverage and poor quality of many health facilities. This resulted in an increased need for health reform, increasing public health expenditure and pro-poor health care [55,56]. Although adult mortality rate has declined over recent years, it is still relatively high at 186 per 1000 population in 2006 [30].

• Virtuous cycle (high globalization, low mortality): In the Netherlands, mortality started to decrease progressively in the late nineteenth century. Although this decline happened decades before the start of modern-day globalization, the diffusion of knowledge about, for example, sanitation probably played an important role besides improved overall living conditions [8]. Adult mortality rate was 92 per 1000 population in 1990, declining further to 70 per 1000 population in 2006 [30].

The important issue for policy purposes, of course, is how a country may move towards the virtuous cycle and several important research questions can be identified. How have countries changed their location over time and due to which underlying mechanisms? If countries find themselves in a viscous cycle, should they first focus on enhancing their health status or on enhancing their integration into the globalization process? Looking at the health-lopsided countries and the globalisation lop-sided countries, which have a higher chance of reaching a virtuous circle and which are most at risk from shifting to a vicious circle? How can health-lopsided countries make sure that their health status is not compromised by any efforts to improve their integration in the globalization process? How can globalisation-lopsided countries increase their health benefits of globalisation? And finally, will the countries that now experience a virtuous cycle also persist to remain in this category in the future?

What is clear is that the increasing complexity of our global society means that sustainable health cannot be addressed from a single perspective, country, or scientific discipline. Changes in human health in the context of globalization are far more complex than health issues that had to be tackled in the past. As addressed by others (e.g., Borgesi and Vecelli [44]), it is our hope that a further analysis of health impacts of globalization may help in adjusting and optimising the process of globalization on every level in the direction of a sustainable and healthy development [57]. To this end, extensive empirical work is needed to identify the relevant causal mechanisms underlying the influence of globalization on human health.

## Competing interests

The authors declare that they have no competing interests.

## Authors' contributions

PM and MR developed the MGI; SA, MH and PM participated in the design of the study and performed the statistical analysis. All authors read and approved the final manuscript.

## Appendix

i The variables excluded from the analysis based on these reasons are: from WHOSIS [30,31]: adult literacy rate (%); adolescent fertility rate (%); antenatal care coverage - at least four visits (%); births attended by skilled health personnel (%); prevalence of HIV among adults aged ≥15 years (per 100 000 population); population with sustainable access to improved drinking water sources (%) total; population with sustainable access to sanitation (%) total; prevalence of current tobacco use amongst adolescents (13-15 years (%) both sexes; prevalence of current tobacco use amongst adults (≥15 years) (%) both sexes; deaths amongst children under 5 years of age due to malaria (%); deaths due to HIV/Aids (per 100 000 population per year). Confounders assessed and excluded for the same reasons from the World DataBank [36] include: malnutrition prevalence, weight for age (% of children under 5); literacy rate adult female (% of females ages 15 and above); literacy rate adult male (% of males ages 15 and above); total enrolment, primary, female (% net); total enrolment, primary, male (% net); pregnant women receiving prenatal care (%); and births attended by skilled health staff (% of total).

ii Variables that did not satisfy the criteria of functioning as a confounder on the MGI-health indicator relationships are: 'Smoking prevalence, males (% of adults) 2006'; and 'Prevalence of HIV, total (% of population ages 15-49), 2007' [36]

iii The following tests have been used to assess whether the indicators used display a normal distribution: Frequency histograms (for a graphical assessment of normality of distribution); P-P plots and Q-Q plots (have been used as a complementary graphical assessment tool for the normality of the distribution of the variable, thus in addition to the frequency histograms); Boxplots (to graphically check for outliers and skewness); the Shapiro-Wilk's W-test (as a formal test for normality has been used[37]. However, results of the W-test have been treated with care and placed within the context of the insights gained from all the other normality tests performed); descriptive statistics have been used to numerically assess skewness and kurtosis (criterion used for skewness: the skewness-statistic must lie between +2 and -2; criterion used for kurtosis: the kurtosis-statistic must lie between +2 and -2)[38].

iv All assumptions of least squares regression analysis have been checked and could be met by the models. The assumption of linearity has been checked with scatterplots and linear curve estimation. The normality of the probability distribution of the error terms of prediction have been tested by generating frequency histograms of the standardized residuals. To test for homoscedasticity, the standardized residuals and the standardized predicted values have been plotted in a scatterplot to observe a random pattern. For the assumption of mean independence, residual statistics and scatterplots of the residual against the predicted values have been used to verify that the mean of the residuals would be approximately zero. In addition, all models have been checked for multivariate outliers by generating Cook's Distances[58]. When the Cook's Distance is higher than 1.0, a case is considered an outlier and is deleted from the analysis.

v Note: The step-wise backward linear regression analyses have been performed manually.

vi When reporting on statistical results, the term 'significance' refers to 'statistical significance'.

vii 'GDP per capita growth (annual%) 2008'; 'Health expenditure, public (% of GDP), 2007'; 'Prevalence of undernourishment (% of population) 2006)'; Immunization, measles (% of children ages 12-23 months, 2008'; 'Improved water source (% of population with access) 2000-2006'; 'Total enrollment, primary (% net) 2000-2008'; 'Smoking prevalence, females (% of adults) 2006'; 'Literacy rate, adult total (% of people ages 15 and above) 2000-2008'; 'Total fertility rate (per woman), 2006'.
